# An up-date on the prevalence of sickle cell trait in Eastern and Western Uganda

**DOI:** 10.1186/1471-2326-10-5

**Published:** 2010-06-23

**Authors:** Andrew L Okwi, Wilson Byarugaba, Christopher M Ndugwa, Arthur Parkes, Michael Ocaido, James K Tumwine

**Affiliations:** 1 Department of Pathology, Makerere University, College of Health Sciences, P.O. Box 7072, Kampala, Uganda; 2 Directorate of Postgraduate Studies and Research, Kampala International University Western Campus P.O. Box 71 Ishaka, Uganda; 3 Department of Paediatrics and Child Health, Makerere University, College of Health Sciences, P.O. Box 7072, Kampala, Uganda; 4 Centre for Endocrine and Diabetes Sciences, Cardiff University, Cardiff CF14 4XN, UK; 5 Department of Wildlife and Animal Resources, Faculty of Veterinary Medicine, Makerere University, P.O. Box 7062, Kampala, Uganda

## Abstract

**Background:**

The first survey on sickle cell disease (SCD) done in Uganda in 1949, reported the district of Bundibugyo in Western Uganda to have the highest sickle cell trait (SCT) prevalence (45%). This is believed to be the highest in the whole world. According to the same survey, the prevalence of SCT in the districts of Mbale and Sironko in the East was 20-28%, whilst the districts of Mbarara and Ntungamo in the West had 1-5%. No follow-up surveys have been conducted over the past 60 years. SCA accounts for approximately 16.2% of all pediatric deaths in Uganda. The pattern of SCT inheritance, however, predicts likely changes in the prevalence and distribution of the SCT. The objective of the study therefore was to establish the current prevalence of the SCT in Uganda.

**Methods:**

This study was a cross sectional survey which was carried out in the districts of Mbale and Sironko in the Eastern, Mbarara/Ntungamo and Bundibugyo in Western Uganda. The participants were children (6 months-5 yrs). Blood was collected from each subject and analyzed for hemoglobin S using cellulose acetate Hb electrophoresis.

**Results:**

The established prevalence of the SCT (As) in Eastern Uganda was 17.5% compared to 13.4% and 3% in Bundibugyo and Mbarara/Ntungamo respectively. 1.7% of the children in Eastern Uganda tested positive for haemoglobin ss relative to 3% in Bundibugyo, giving gene frequencies of 0.105 and 0.097 for the recessive gene respectively. No ss was detected in Mbarara/Ntungamo.

**Conclusions:**

A shift in the prevalence of the SCT and ss in Uganda is notable and may be explained by several biological and social factors. This study offers some evidence for the possible outcome of intermarriages in reducing the incidence of the SCT.

## Background

Sickle cell disease is an inherited hemoglobinopathy arising from the substitution of glutamic amino acid by valine in the sixth position of the beta globin chain [1]. Inheritance of the sickle cell trait follows a recessive autosomal pattern. Phenotypically, only persons with double recessive genes of sickle cell (ss homozygotes) do manifest disease, whilst the heterozygotes (AS) are being referred to as carriers. According to Diallo, Africa is the most highly affected continent with 200,000 new born affected by sickle cell anemia (SCA) per year [1]. This constitutes approximately 66.6% of the children born with haemoglobinopathies worldwide. According to reports from Ghana, it is estimated that 15,000 children are born with sickle cell disease (SCD) annually [2]. In Benin, the sickle cell trait (SCT) prevalence is estimated to be 25% [3] while in Nigeria it ranges from 24-25% [4,5]. The prevalence of the sickle cell gene among the Chagga tribe in Tanzania on the other hand is about 4%. In all these African countries, the concentration of the SCT has been found to be highest in specified sub-populations [1-5], likely due to tribal conservative marriages. The autosomal recessive pattern of inheritance assumed by SCT, however, predicts changes in population-wide dynamics, ease of movement and inter-tribal marriages which would alter the SCT distribution within these communities [6].

The available data on the population-wide prevalence of SCD in Uganda is based on a survey by Lehmann and Raper in 1949 [7]. According to the same, the prevalence of SCT among Ugandan tribes, ranged from 4 to 45%, and was reported to be the highest among the Baamba in the West where malaria is endemic [7]. Of the 900,000 thousand children born annually in Uganda [8], approximately 2.8% have sickle cell anemia (ss homozygotes) [9]. A staggering 20,000 (70-80%) of these children born with SCA presumably die before their 5^th ^birth day. This number contributes 16.2% of all children (123,000) who die annually in Uganda [10].

In light of the advances in care interventions, pre-marital genome-wide population screening and counseling, it is supposed that the distribution of the SCT among African populations may be strategically altered. However, evidence for positive outcome of intermarriage in reducing the incidence of the SCT needs to be established. We hypothesized that, a current survey of the prevalence of sickle cell disease in Uganda, when related to prior findings by Lehmann and Raper [7], will be informative of the same. This study was therefore conducted to ascertain an up-date on the prevalence of sickle cell trait in Eastern and Western Uganda.

## Methods

### Study design

This was a cross sectional survey.

### Study populations and sites

**Figure 1:**
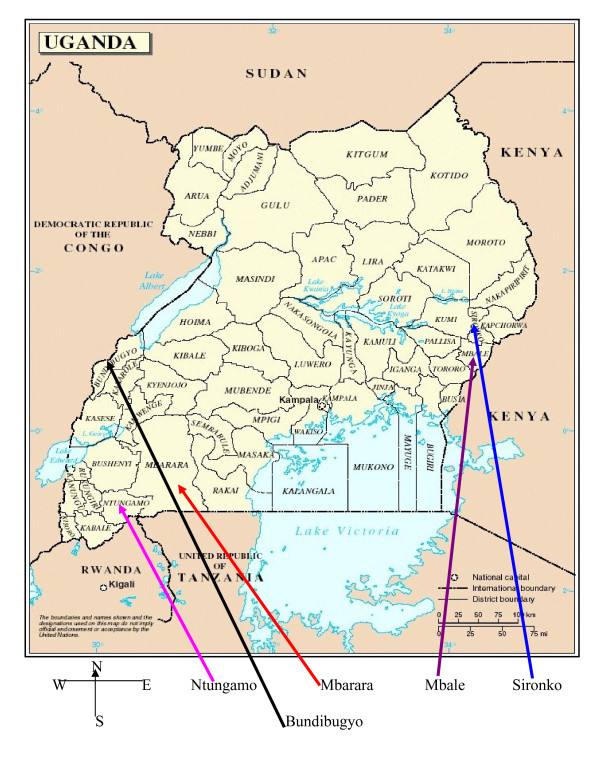
**Map of Uganda, showing location of study districts**. This map aims to orientate the reader in regard to locations of the major study districts:Mbale and Sironko (East); and Mbarara/Ntungamo and Budibugyo (West).

We studied populations in five districts: Sironko and Mbale in Eastern; Mbarara, Ntungamo and Bundibugyo in Western Uganda (See figure 1 for a map of Uganda and location of the study districts). The district of Bundibugyo was selected because of its uniqueness in that it is believed to have the highest prevalence (45%) of sickle cell trait in the world [7].

### Study subjects

These were children aged between 6 months and 5 years. The sample size of 571 was calculated using Kish and Leshlie formula [11]. The prevalence of 23.8% was used for eastern, 5% for Mbarara/Ntungamo and 45% for Bundibugyo in Western Uganda as established by Lehman. The precisions of 5% and 2.5% were used for Eastern and Mbarara and Ntungamo respectively, while a precision of 7% was taken for Bundibugyo. A confidence interval of 95% was used. Through-out the recruitment, selection of participating children was done randomly, using a register of populations at the local council I level. Specifically, serial recruitment was done using odd numbers viz: 1, 3, 5, 7...,13,15.

This study was approved by the Makerere University College of Health Sciences (formerly faculty of Medicine) Research and Ethics Committee. For all the participating children, a written informed consent was obtained from legal authority (guardian or parent).

### Sample collection

A maximum of 2 mls of blood sample was collected in ethylene diamine tetra acetic acid (EDTA) vacutainers from either a hand vein or by heel puncture from each infant at health centers and nursery schools. Samples were delivered in ice boxes to Mulago hospital for analysis using Hb electrophoresis at pH 9.2 on cellulose acetate strips [12]. Hb electrophoretic bands were then stained and compared with the known controls.

• Between March and August 2007, a total of 656 blood samples were collected from 656 randomized children in Male and Sironko in Eastern; and Mbarara and Ntungamo in the West which was a little (1.1%) more than the minimum calculated sample size of 571. A total of 286 and 370 children participated in the study from Eastern and Western respectively.

• From June to July 2009, a total of 201 blood samples were collected from 201 children in Bundibugyo district in Western Uganda which was (1.0%) more than the calculated sample size of 194

### Data analysis

The data was entered and analyzed by using statistical package for social sciences (SPSS 10.0) SPSS Advanced Statistics 10.1. Chicago: SPSS Inc, 2000). The gene frequency was calculated using the Hardy Weinberg Law [13]. The statistical difference of the prevalence of SCT and SS in these study populations was compared using Open Source Epidemiologic Statistic Program for Public Health version 2.2.1 (OPENEPI) using 2 × 2 contingency tables [14]. A p value of ≤0.05 was considered statistically significant.

## Results

**Figure 2:**
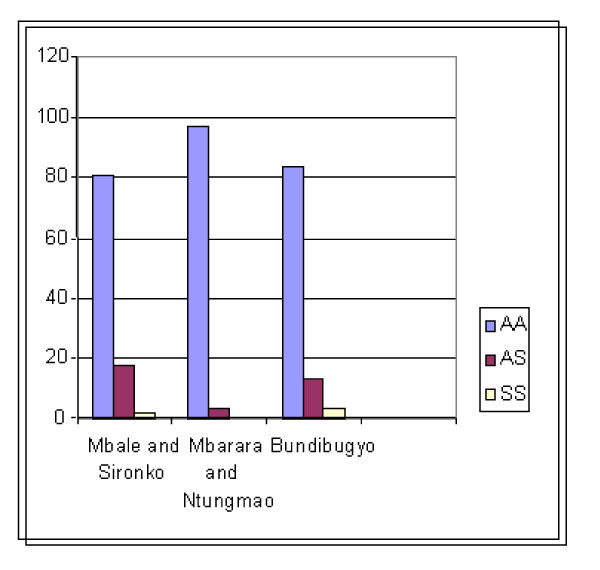
**The percentage of haemoglobin A, AS and SS detected in our study population**. This data describes the percentage of haemoglobin A, AS and SS detected in our study population. The districts of Uganda represented are Mbale and Sironko (East); and Mbarara/Ntungamo and Budibugyo (West).

In Mbale and Sironko districts, 231 children (80.8%) were positive for Hb AA, 50 (17.5%) for Hb AS and 5 (1.7%) for SS, while Mbarara and Ntungamo districts had 359 children positive for Hb AA (97%), 11 for Hb AS (3%) and none had SS. In Bundibugyo district, 168 were positive for AA (83.6%), 27 for AS (13.4%) and 6 (3%) for SS (see Table 1 and Figure 2).

**Table 1:** Summary of the observed prevalence of AS and SS and expected prevalence of SS in eastern and western Uganda, 2007.

Study area	Observed prevalence of AS (%)	Observed prevalence of SS (%)	Expected prevalence of SS*
**Mbale/Sironko (Eastern Uganda)** **n = 286**	17.5	1.75	4.20
	[95% CI 13.5-22.3]	[95% CI 0.63-4.15]	
**Mbarara/Ntungamo (Western Uganda)** **N = 370**	3.0	0.0	1.70
	[95% CI 1.61-5.31]	[95% CI 0.0-1.24]	
**Buundibugyo (Western Uganda)** **N = 201**	13.4	3	3.70
	[95% CI 9.35-18.94]	[95% CI 0.31-4.50]	

* Expected prevalence of SS = square root of AS prevalence according to Mordell and Darlison [16].

In Eastern Uganda, with a heterozygous prevalence of 17.5% and homozygous prevalence of 1.7%, the total percentage of abnormal genes within the population was calculated to be ([17.5/2 +1.7]) or simply 10.45%. This yields a gene frequency of 0.105. The expected gene frequency of the homozygotes in Eastern Uganda, given a gene frequency of 0.105, would be (0.105) [2] or 1.1% In Western Uganda (specifically Bundibugyo), at a heterozygous prevalence of 13.4% and homozygous prevalence of 3%, the total percentage of abnormal genes within the population was ([13.4/2 +3]) or simply 9.7%. This yields a gene frequency of 0.097. The expected gene frequency of the homozygotes in Bundibugyo, given a gene frequency of 0.097, would be (0.097) [2] or 0.94%.

As a notable finding, we observed that the statistical difference in the prevalence of AS between Mbarara/Ntungamo in the West and both Mbale/Sironko in the East and Bundibugyo in the West was highly significant (<0.001). There was however no statistical difference in the prevalence of AS between Mbale/Sironko in the East and Bundibugyo in the West (>0.05). The difference in the prevalence of homozygous state (SS) of sickle cell disease between Bundibugyo in the West and Mbale/Sironko in the East was statistically insignificant (>0.05). The statistical differences in the prevalence of AS and SS between the study districts is as shown in Table 2.

**Table 2:** The statistical difference in the prevalence of AS and SS between the study districts.

Variable	Mbale and Sironko	Mbarara and Ntungamo	Odds Ratio	CI	P-value
AS	50 (286)	11 (370)	6.91	3.53-13-13.6	0.001
Variable	Mbale and Sironko	Bundibugyo	Odds Ratio	CI	P-value
AS	50 (286)	27 (201)	1.36	0.82-2.27	0.110
SS	5 (286)	6 (201)	0.58	0.17-1.92	0.193
Variable	Bundibugyo	Mbarara and Ntungamo	Odds Ratio	CI	P-value
AS	27 (201)	11 (370)	5.06	2.46-10.45	0.001

## Discussion

Contrary to earlier report by Lehmann and Rapper [7], our results show that, the prevalence of the AS carrier state was highest in Eastern Uganda, followed by Bundibugyo (see Figure 1-map of Uganda, and Figure 2 for distribution of results). The fact that the first survey on sickle cell disease (SCD) in Uganda was done over 60 years ago, and no similar surveys have been conducted since then; it is not surprising that SCD has changed its dynamics. This is because the inherited nature of the SCD trait predicts likely changes in the prevalence and distribution of the same depending on the prevailing biological (malaria interventions) and social factors (marriage patterns). Indeed, contrary to an earlier report of 45% prevalence of the AS trait in Bundibugyo, we found the current AS prevalence to be 13.4%; changes that we have appropriated to the possible earlier adoption of intermarriage-avoidance in Bundibugyo resulting from a similarly earlier knowledge of AS prevalence [7]. Specifically, although the difference in the prevalence of AS between Eastern Uganda and Bundibugyo in the west was statistically insignificant, the prevalence of AS in Bundibygyo (13.4%) was found to be much lower than estimated by Lehmann and Raper (45%), see Tables 1 &2 for details. Several hypotheses may be used to explain this finding. The same should, however, be cautiously employed here since huge heterogeneity within the spatial distribution of genetic factors have been found in many studies (even over relatively very small areas) and the small number of subjects sampled in our study makes such comparisons inaccurate. First, basing on the first survey of 1949, which was 60 years ago, the Baamba was one of the exclusively preserved tribes in Uganda which practiced high level of consanguinity. However, due to the movement of the people, the Baamba may have intermarried into other tribes leading to sickle cell gene admixture or dilution. Secondly, though it is possible that improved malaria interventions may have selected for increased numbers of haemoglobin AA and decreased numbers of AS [15], it is unlikely, because the heterozygous HbAS trait has been observed to have beneficial effects in protecting against severe forms *P. falciparum *infection. On the other hand the prevalence of SCT in Mbale/Sironko was slightly lower than the 20-28% estimated by Lehmann) and Rapper [7]. It is possible that due to improved malaria intervention programmes in Uganda, some of the people with AS may have been denied resistance against malaria infection as already explained above.

A number of notable difference between our and Lehmann and Raper's study [7] are observable. For instance, while our finding of 3% AS in Mbarara/Ntungamo was similar to the 1-5% reported by Lehmann, it is clear that the prevalence of AS in these districts had remained low. The most likely reason for this observation was as cited above. Improved malaria interventions could have denied persons with AS protection against malaria thus keeping their numbers low [15]. Secondly, it is possible that the level of intermarriage between these communities and other tribes could still be very low and as a result, emergence of new cases of sickle cell disease due to gene admixture may have been curtailed. On the other hand, in as much as the prevalence of SCT in Bundibugyo was lower than 45% reported by Lehmann, it is possible that the prevalence of sickle cell trait among Baamba adult population is still high. This hypothesis is supported by the fact that the study found high prevalence of SS among the children which could be reflective of a high prevalence of AS in the adult population. This finding is further supported by the fact that our study sampled children while Lehmann study sampled adults. The observed prevalence of SS in all these districts was actually much lower than expected [16]. In absence of cost effective screening interventions [17] and therapeutic measures for sickle cell disease within this setting such as bone marrow transplantation and gene therapy [18], the obvious question to ask regards what may have happened to the missing SS progeny. It is possible that many of these children could have succumbed to the disease before celebrating their fifth birthday because of the absence of comprehensive sickle cell screening and management programmes in these districts. Serjeant and Ndugwa alluded to this in their advocacy paper [9]. This hypothesis appeared to have been supported by the fact that all the children who were detected with SS in Mbale/Sironko in Eastern and Bundibugyo in the West were less than 4 years old; suggesting that ss cases are hardly detectable at 5 years since many of these children could possibly have died before their fifth birthday. This may probably further explains why the data on median survival of persons with sickle cell anemia in many developing countries including Uganda is scarce. The fact that no children with SS were detected in Mbarara/Ntungamo does not mean that these children do not exist. The probable reason for this observation was that the number of AS persons may have been kept further low in these districts by natural law of selection as new effective malaria intervention progammes have been put in place This, therefore, could have in turn kept the number of SS low and therefore influencing the detectability of SS cases. Another reason for this could be that the delectability power (precision) used was probably not sensitive enough such that, if this study was to be repeated today using a more sensitive power, children with SS would have been detected in Mbarara/Ntungamo and more children with SS would have been detected in Bundibugyo in the West and Mbale/Sironko in the East. Lastly, there are several shortcomings in our study. First, although we used Hb electrophoresis as our 'gold standard' because of its affordability and availability, better gold standards such as automated capillary Hb electrophoresis are preferred. (Sebia Parc Technlonogique, Leonard de Vinci. CP 8010 Lisses-91008. EVRY Cedex-France). Secondly, in as much as the recruitment of the study participants was randomized, the selection of the study districts was based on convenience sampling and did not therefore give equal chance to the rest of the districts in Eastern and Western Uganda to be represented. So the current results cannot be generalized as representative of the whole Eastern or Western Uganda. Thirdly, as observed above (results section), the statistical difference in the prevalence of AS between Mbarara/Ntungamo in the West and both Mbale/Sironko in the East and Bundibugyo in the West was highly significant (<0.001); and this may affect our study versus real figures on ground.

## Conclusion

Contrary to earlier reports, our study suggests the possible decline in the prevalence of the AS carrier state especially amongst the Baamba of western Uganda. This shift might have resulted from several social (marriage patterns) and possibly biological (malaria interventions) factors.

## Competing interests

The authors declare that they have no competing interests.

## Authors' contributions

ALO conceived the idea, participated in data collection, analysis, and writing of the final manuscript.; WB, CMN and MO participated in conception of the idea, supervised the study and data interpretation.; AP participated in data analysis, JKT participated in conception of the idea, supervised the Bundibugyo component of the study, contributed to data analysis and writing of the final manuscript. All authors read and approved the final manuscript.

## Pre-publication history

The pre-publication history for this paper can be accessed here:

http://www.biomedcentral.com/1471-2326/10/5/prepub
